# Supplementary material for: Circulating Insulin-Like Growth Factor I is Involved in the Effect of High Fat Diet on Peripheral Amyloid β Clearance
Source: Int J Mol Sci. 2020 Dec 18;21(24):9675. doi: 10.3390/ijms21249675 (PMC7766006; doi:10.3390/ijms21249675)
Supplement: Supplementary file 1 [file ijms-21-09675-s001.pdf]

International Journal of *Molecular Sciences*

MDPI

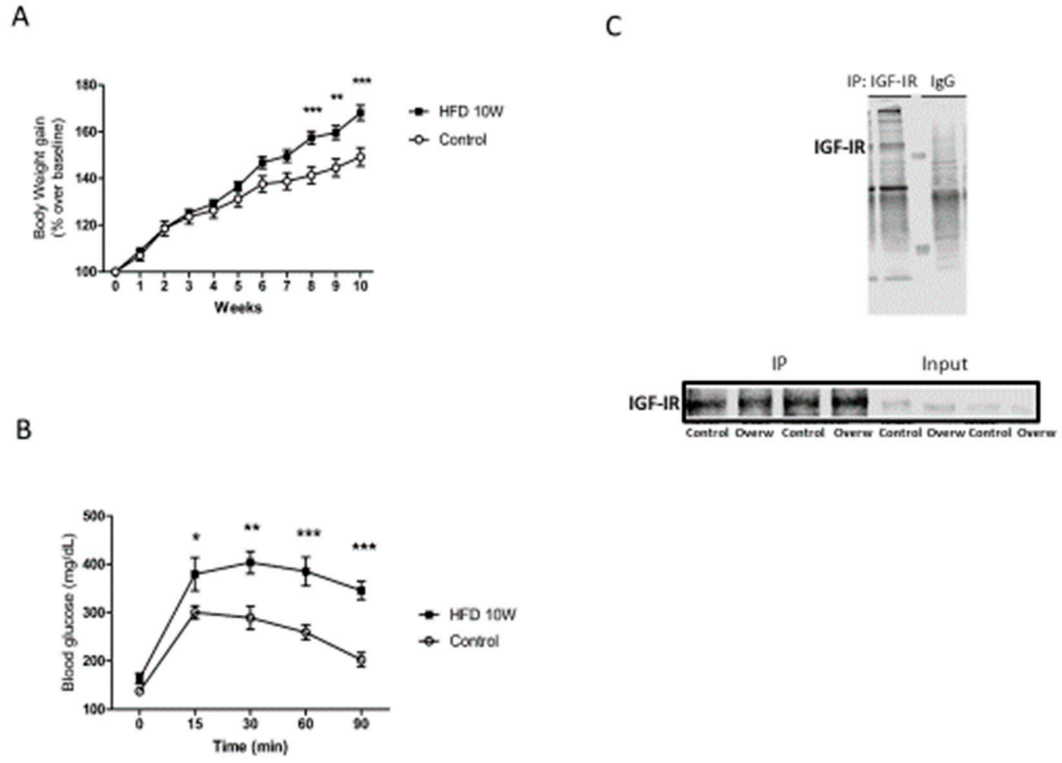

Supplementary Figure S1. A. Mice submitted to a high fat diet during 10 weeks (W) show increased body weight along time (n=10 per group). **B.** Overweight mice show reduced tolerance to systemic glucose load, as determined by significantly greater blood glucose levels over 90 min after glucose administration. **C.** Immunoprecipitation controls. Upper blot: lysate was immunoprecipitated with either anti-IGF-IR or non-immune IgG and blotted with anti-IGF-IR. A band corresponding to IGF-IR is seen only after immunoprecipitation with anti-IGF-IR. Lower blot: lysates from control and overweight (overw) brains were immunoprecipitated with anti-IGF-IR (IP) or directly assayed by IGF-IR western blot (input).
